# Effects of physical exercises on inflammatory biomarkers and cardiopulmonary function in patients living with HIV: a systematic review with meta-analysis

**DOI:** 10.1186/s12879-019-3960-0

**Published:** 2019-04-29

**Authors:** S. C. Ibeneme, C. Omeje, H. Myezwa, Salome Nwaelom Ezeofor, E. M. Anieto, F. Irem, Amaka Obiageli Nnamani, Fortune Elochukwu Ezenwankwo, G. C. Ibeneme

**Affiliations:** 1 Department of Medical Rehabilitation, Faculty of Health Sciences, University of Nigeria, Enugu Campus, Enugu, Nigeria; 2 Department of Physiotherapy, Faculty of Health Sciences, School of Therapeutic Studies, University of the Witwatersrand, 7 York Road, Parktown, Johannesburg, 2193 South Africa; 3 Department of Radiation Medicine, Faculty of Medical Sciences, College of Medicine, University of Nigeria, Ituku-Ozalla Campus, Enugu, Nigeria; 4 Department of Nursing Sciences, Ebonyi State University, Abakaliki, Ebonyi State Nigeria; 5 Clinical Trial Consortium University of Nigeria, Nsukka, Nigeria

**Keywords:** Physical exercises, Aerobic exercises, Resistance exercises, Human immunodeficiency virus, Patients living with HIV, Highly active antiretroviral therapy, Inflammatory biomarkers, Cardiopulmonary function, Quality of life, Systematic review, Meta-analysis

## Abstract

**Background:**

Pro-inflammatory cytokines expressed in human immune deficiency virus (HIV) infection, may induce oxidative stress likely to compromise the patency of the airways or damage the lung tissues/cardiac function. However, physical (aerobic and/or resistance) exercise-induced release of heat shock protein, immune function alteration or reduced tissue hypoxia, have been highlighted as possible mechanisms by which increasing physical activity may reduce plasma pro-inflammatory cytokines in uninfected individuals and should be appraised in the literature for evidence of similar benefits in people living with HIV (PLWH). Therefore, we evaluated the effects of physical exercises on 1) inflammatory biomarkers and 2) cardiopulmonary function (VO_2_ Max) in PLWH.

**Method:**

A systematic review was conducted using the Cochrane Collaboration protocol. Searching databases, up to January 2018. Only randomized control trials investigating the effects of either aerobic or resistance or a combination of both exercise types with a control/other intervention(s) for a period of at least 4 weeks among adults living with HIV, were included. Two independent reviewers determined the eligibility of the studies. Data were extracted and risk of bias (ROB) was assessed with the Cochrane Collaboration ROB tool. Meta-analyses were conducted with random effect models using the Review Manager (RevMan) computer software.

**Result:**

Twenty-three studies met inclusion criteria (*n* = 1073 participants at study completion) comprising male and female with age range 18–65 years. Three meta-analyses across three sub-groups comparisons were performed. The result showed no significant change in biomarkers of inflammation (IL-6 and IL-1β) unlike a significant (Z = 3.80, *p* < 0.0001) improvement in VO_2_ Max. Overall, the GRADE evidence for this review was of moderate quality.

**Conclusion:**

There was evidence that engaging in either aerobic or resistance exercise, or a combination of both exercises, two to five times per week can lead to a significant improvement in cardiopulmonary function but not biomarkers of inflammation (IL-6 and IL-1β). However, this should not be interpreted as “No evidence of effect” because the individual trial studies did not attain sufficient power to detect treatment effects. The moderate grade evidence for this review suggests that further research may likely have an important impact on our confidence in the estimate of effects and may change the estimate.

**Electronic supplementary material:**

The online version of this article (10.1186/s12879-019-3960-0) contains supplementary material, which is available to authorized users.

## Background

For decades, the Human Immunodeficiency Virus (HIV), which induces cell death (CD4- helper cells) [1], has remained a key public health issue globally [2]. Global epidemiological data suggest that HIV-related deaths are common among women in the reproductive age group (15–49), especially the Sub-Saharan Africa which accounts for 64% of the total new cases of HIV infection [3]. Meanwhile, Nigeria is the second largest HIV-prevalent country in the world, after South Africa [4]. However, the discovery of the Highly Active Antiretroviral therapy (ART) and its advancement has changed the severity of the HIV infection, for which reason it is currently managed as a chronic condition [5], rather than an outright “death sentence.” Thus, ART usage reduces the mortality rate associated with HIV by decreasing the viral load, boosting the immune function, and reducing the threat of AIDS-defining opportunistic illnesses (OIs) [6]. A reduction in opportunistic diseases was possible through a reduction of HIV-related inflammation/immune activation [7], decreased risk of HIV transmission [8], improved CD4 T-cells/immune response and therefore decreased progression to AIDS [9]. All these effects of ART mentioned above account for a remarkable improvement in the life expectancy of the people living with HIV (PLWH) [10]. However, despite the benefits of this drug, PLWH are still associated with lactic acidosis, dyslipidemia & lipodystrophy, neuropsychiatric symptoms, liver toxicity, gastrointestinal intolerance, lipid abnormalities [11], increase creatinine kinase level [12] and cardiopulmonary dysfunction [13]. Some of these conditions exist as a result of an increased inflammatory response associated with HIV [14, 15] which is not fully suppressed by the use of ART [16–18]. Hence, high levels of Interleukin 18 (IL-18) in the sputum/plasma and plasma-related Interferon gamma (IFN-ϒ), which is associated with high pressures from the pulmonary artery [19], have been reported in PLWH. Therefore, early management of inflammation is essential in tempering HIV/ ART -associated morbidity [20], and can be achieved through improved physical activity level.

Systemic inflammation commonly associated with HIV is linked to many chronic diseases [21]. In fact, increased expression of pro-inflammatory mediators may trigger the release of reactive oxygen species (ROS) and constitutes an important component of molecular and cellular tissue damage mechanisms in a wide range of chronic human diseases [22, 23] that may include cancer and cardiopulmonary conditions. In fact, the C-reactive protein (CRP – an acute phase protein and a biomarker of inflammation) is increasingly considered to be involved In systemic response to inflammation [24], and lies on the path of biological plausibility for cancer induction [25]. Similarly, several studies had observed high levels of systemic fibrinogen and CRP with pulmonary dysfunction [14], especially; chronic obstructive pulmonary disease [26]. For instance, there is evidence of an association between circulating inflammation markers and forced expiratory volume at the first second (FEV_1_) [27], though the physiological mechanisms relating low-grade systemic inflammation and pulmonary disease is not fully delineated [28, 29]. However, it is possible that the induced oxidative stress would have involved pro-inflammatory mediators, which should adversely compromise the patency of the airways or damage the lung tissues/cardiac function [30, 31]. Therefore, factors that influence systemic inflammation may play a role in the induction of cardiopulmonary diseases in HIV conditions and should be addressed in prevention efforts. Physical exercise has been reported as having anti-inflammatory effects in intervention and large population studies of non-HIV infected individuals [32, 33], and therefore may attenuate inflammatory response in HIV conditions.

Physical exercise intervention has resulted to a significant increase in cardio-pulmonary fitness [34] and improved quality of life (QoL) [35] mostly linked to improved aerobic capacity, endurance [36] and immune function (i.e. improved CD^4+^ cell counts) in PLWH [37]. In addition, a number of studies propose that a lifestyle of physical activity in moderate/high intensities reduces the serum concentration of C-reactive protein (CRP) by 6–35% [38] and improves cardiorespiratory fitness [39]. Invariably, these findings suggest that the profile of serum concentration of CRP and cardiorespiratory fitness may vary proportionately to the level of physical activity. Hence, a physically active lifestyle may not only be expected to improve chronic inflammation, (which have been implicated in the induction of cancer) but likewise cardiorespiratory fitness/function, which should positively influence the quality of life (QoL). This view was corroborated by findings from the Women’s Health Study (WHS) prospective study of 27,055 healthy elderly women, which revealed that physical activity ameliorated the risk of cardiovascular disease (CVD) through its effect on chronic inflammation and other known risk factors [40]. In addition, other studies provided valuable insights into the direct effects of aerobic and resistance exercise training on certain health indices often compromised by HIV infection and ART. For instance, Levinger et al. [41], and Philips et al. [42], indicated that resistance training has positive effects on chronic diseases by reducing the plasma concentrations of pro-inflammatory cytokines. Neto et al. [43], also reported that combined aerobic and resistance exercises are effective in improving muscle strength, energy, VO_2_ peak and health status of patients with HIV. Another study by Zanetti et al. [44], demonstrated the efficacy of non-linear resistance training in adjusting the inflammatory profile and anthropometry of PLWH.

So far, the mechanism by which increasing levels of physical activity improve plasma CRP seems to be explained by other factors apart from its effect on body weight, since both low levels of physical activity and high levels of body mass index (BMI) were independently associated with increased serum levels of inflammatory biomarkers [45]. Thus, the exercise-induced release of heat shock protein, immune function alteration or reduced tissue hypoxia, have been highlighted as other possible mechanisms by which increased physical activity may reduce plasma CRP [46]. By itself, physical activity may attenuate systemic inflammation or when combined with relevant changes in body weight or composition [47]. Moreover, there is evidence that physical activity boosts the activity of macrophages, natural killer cells, lymphokine-activated killer cells, neutrophils, and regulating cytokines with net anti-inflammatory and immunomodulation effects, which may contribute to the protective value of physical activity [48]. Based on the available scientific evidence so far cited, it is reasonable to expect that physical exercise will improve or sustain cardiopulmonary/physical fitness and general health and wellness [49], in HIV conditions. Therefore, it is understandable why physical exercise has been proposed as an adjunctive therapy for drug intervention in people with chronic diseases [50].

There is no doubt that the literature is replete with scientific evidence on the positive effects of physical exercises on multisystemic function in PLWH, and is therefore considered suitable as part of their management program [51]. Although, the results of some studies suggest an association between high physical activity level and lower levels of C-reactive protein and other surrogate markers of inflammation, findings from intervention studies of exercise alone or exercise and diet combined have been conflicting [52, 53].. Accordingly, the effects of physical activity on surrogate biomarkers of systemic inflammation, especially CRP, in patients with HIV may still be faced with much debate. Therefore, notwithstanding the positive findings in support of the role of physical exercise modalities in ameliorating symptoms of systemic inflammation, and cardiopulmonary function, a systematic appraisal of the available evidence in the literature is still required to determine the level of evidence, and therefore guide practice and further research. The aim of this systematic review was therefore to determine the impact of physical exercises on cardiopulmonary function and biomarkers of inflammation in HIV patients. The review question is: What are the effects of physical exercises (aerobic exercises or resistance exercises) on cardiopulmonary function and Inflammatory biomarkers in patients living with HIV? To answer the review question, specific review objectives sought to determine the effects of physical exercises on 1) inflammatory biomarkers and 2) cardiopulmonary function in patients living with HIV.

## Methods

This Systematic review used the International Prospective Register of Systematic Reviews (PROSPERO) criteria and was certified on 21st June 2018 (registration number PROSPERO 2018: CRD42018098667).

### Eligibility criteria

Eligibility criteria considered for selecting studies in the review include:

Inclusion criteria:

1. Type of studies
  Original research manuscripts in peer-review journals and conferences proceeding were included if published in the English Language. The review design for this study included RCTs and case-control studies, whose objective(s) included the evaluation of the: effects of physical exercises (aerobic/resistance) on inflammatory biomarkers and associated cardiopulmonary function in PLWH that are on ART treatment.
2. Type of participants
  The review included studies of exercise intervention done on patients infected with HIV. Studies involving adult human participants aged ≥18 years. No specific limitation was considered with respect to the study setting of the included studies. The studies included were mainly carried out in clinics, health centres, hospitals or community care settings.
3. Type of intervention
  RCTs and case-control trials of physical exercise intervention for PLWH were included in the review, which was not restricted to specified dosage, form, intensity, frequency and duration of intervention or follow-up period after aerobic intervention nor limited to weight training, isometric and isotonic strengthening for resistance exercise in PLWH.
4. Type of control
  RCTs and case-control trials of PLWH who were placed on medications for HIV treatment (ART) with/without counselling was used. The medical treatment was limited to any particular type of ART drug.
5. Timing
  Studies were included only if outcome assessment were conducted at the completion of the intervention and/or at ≤6 months post-intervention.
6. Types of outcome measures
  Studies that reported on observable changes in these or any other outcome measure of inflammatory biomarkers/cytokines - CRP, high sensitivity C-reactive protein (Hs-CRP), interleukin-1β (IL-1β), Interleukin-2(IL-2), interleukin-4 (IL), interleukin-5 (IL-5), Interleukin-6 (IL-6), Interleukin-8 (IL-8), Interleukin-10 (IL-10), Interleukin 18 (IL-18), Tumour necrosis factor alpha (TNF- α), Interferon gamma (IFN-ϒ), D-dimer, Soluble Tumor Necrosis Factor-II Receptor, soluble CD14(sCD14) and cardiopulmonary function - Peak Oxygen uptake (VO_2_ peak), VO_2_ max, FEV_1_, Tidal volume (TV), Oxygen pulse (O_2_ pulse), submaximal oxygen uptake (VO2 sub), maximal work rate (Wrmax), peak running speed (Vpeak), the slope/intercept values for heart-Rate workload relationship, 5 min Submaximal analysis of HR (HR_5min_) and ventilation (VE).

Outcome measures were not categorised into primary or secondary outcomes. Only studies involving inflammatory or cardiopulmonary outcomes were included. Analysis of clinical results to obtain effects of physical exercise intervention on inflammatory or cardiopulmonary outcomes was undertaken and graded into various levels of evidence using the Grading of Recommendations Assessment Development and Evaluation (GRADE) approach.

Exclusion criteria:

1. Studies without an exercise or physical activity component.
2. Narratives review synthesis, systematic reviews, opinion papers, letters and any publication without primary data and/or explicit description of the methods.
3. Duplicate publications from the same study, the most recent or most comprehensive publication.

### Information sources and search strategy

A search strategy was formulated and piloted as shown in Additional files 1 and 2 and based on the guidelines of the Cochrane Handbook for Systematic Reviews [54] and advice for Health Care Review by the Centre for Reviews and Dissemination [55]. This was further adapted for use in other databases (Additional files 3 and 4). Databases included – [Cumulative Index to Nursing and Allied Health Literature (CINAHL), the Cochrane Library, Excerpta Medica dataBASE (EMBASE), Allied and Complementary Medicine Database (AMED), and PubMed]. Trial registers and directory of open-access repository websites were searched using controlled vocabularies and keywords: HIV/AIDS, Seropositive, aerobic exercises, resistance exercises, strengthen exercises, physical exercises, exercise program, exercise intervention, inflammatory biomarkers, cardiopulmonary function. A hand search was performed from the reference lists of identified studies.

### Study record, selection process, and data management

Literature search results were exported into RefWorks to check for duplication of studies and subsequently exported from RefWorks into Microsoft Excel [56] to facilitate the management and selection of articles for inclusion. Eligibility questions and structures for the screening of the studies included in the reviewer were then developed, piloted and subsequently refined. The first review independently screened title, abstract and full texts of selected studies for eligibility and subsequently, a second reviewer validated the results based on the review eligibility criteria. Any difference in opinion regarding inclusion and exclusion was discussed with a third reviewer.

### Quality appraisal and risk of bias

Adopting the Physiotherapy Evidence Database (PEDro) quality appraisal tool [57], the risk of bias for each of the included studies were evaluated by two reviewers using the PEDro 11-item scale in which the first item relates to external validity and the other ten items assess the internal validity of a clinical trial.

The procedures undertaken to assess each item for each study was explicitly described and rated as 9–10 (excellent); 6–8 (good); 4–5 (fair); < 4 (poor). Articles that met the criterion were awarded points. A score of one was given for each yes answer and zero for no, unclear and not applicable (N/A) answers for each study were tallied for all yes answers out of 10 based on the scores of individual items from the critical appraisal tool were added to present the total score. Both reviewers made judgments regarding the risk of bias independent of each other. Areas of differences were resolved by discussion and reflection, or in consultation with the third reviewer.

### Data item

Data was collected from variables including authors’ references, participants’ characteristics, inclusion and exclusion criteria, study sample size, components of the intervention, the intervention setting, who delivered the intervention, the duration of the intervention and follow-up (where available), attrition rate, aspects of outcome assessed, the outcome measurement, methods/techniques, results, conclusions and funding sources.

### Data synthesis and assessment of heterogeneity

To answer the review question regarding the effect of physical exercises on inflammatory biomarkers and cardiopulmonary function in PLWH, all quantitative study outcomes which analyzed the effectiveness of these interventions were considered and presented in a proof table. The appropriate statistical method was used for different variables: for a continuous variable, weighted mean differences were applied when outcomes are uniform or standard mean difference when different outcomes are used with 95%: CI while for a dichotomous variable, the Risk ratio was applied with 95%: CI. This review also includes a meta-analysis to find pooled effect sizes across studies, using a random-effects model relying on the level of heterogeneity of intervention effects. Heterogeneity was assessed through the Cochrane’s χ^2^ test (10% significance level) and Higgins I^2^ for which values of 25, 50, and 75% shows low, medium and high heterogeneity respectively as stipulated by the guidance in the Cochrane Handbook for Systemic Reviews of Interventions [55].

### Data and sensitivity analysis

The primary outcomes were presented. Studies with homogenous characteristics, in terms of design, intervention, and comparator(s), were pooled together and analysed using a random-effects model [54]. Heterogeneous studies were interpreted by narrative synthesis following the guidelines of the Centre for Reviews and Dissemination to explore the relationship and findings between the included studies [55]. The impact of studies with a high risk of bias on the general outcomes was determined using sensitivity analysis.

### Rating quality of evidence and strength of recommendation

The quality of evidence of the studies was evaluated to determine the strength of recommendation in the systematic review. This was judged utilizing the Grading of Recommendations Assessment Development and Evaluation (GRADE) approach [58] which comprise consistency; design; directness; precision; publication bias and study limitations. The individual study was graded as high risk of bias or low risk of bias. Finally, the individual evidence statement for this review was graded from ‘High Quality’ to ‘Very Low Quality’ according to the criteria (Additional file 5).

## Results

### Search result

Two different searches were conducted sequentially during the course of this study using the two-primary outcome separately in the search strategy.

#### Inflammatory biomarkers

**Fig. 1:**
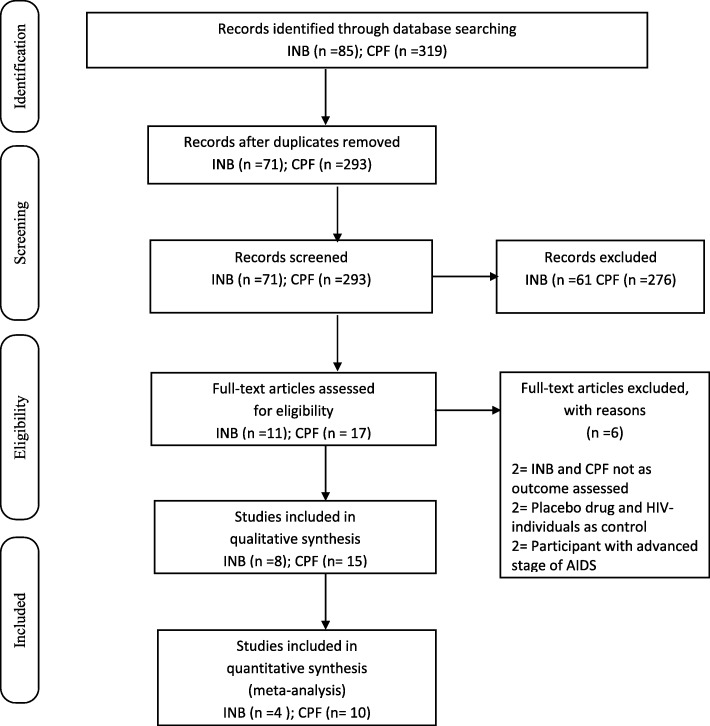
PRISMA Diagram for inflammatory biomarkers (INB), cardiopulmonary function (CPF): adapted from Moher, Shamseer & Clarke, et al., 2015; Preferred Reporting Items for Systematic Reviews and Meta Analyses

Results of searches from all the involved sources produced 85 citations. Out of this number, 14 citations were removed as duplicates and the remaining 71 passed through title and abstract screening that yielded 10 publications. After the full-text screening, 8 articles met the inclusion criteria [44, 59–64]. Figure 1 Preferred Reporting Items for Systematic Reviews and Meta-analyses (PRISMA Flow Diagram).

#### Cardiopulmonary function

Searches in the involved sources resulted in 319 citations. Duplicate screening removed 26 citations and further screening via title and abstract review yielded 18 publications that had to pass through full-text review. After this, 14 articles were considered eligible to be included in the study [36, 65–77].

### Reasons for exclusion

For inflammatory biomarkers; three full texts retrieved were excluded because one of the studies [78] did not access for the required outcome, another [79] involved seronegative individuals as the only control used rather than HIV positive patients and the third study [80] used placebo medication rather than ART.

For Cardiopulmonary function; three articles were excluded for not accessing the required outcome [81], and the inclusion of patients that have reached the advanced stage of AIDS-defining diagnoses [82, 83].

### Included studies

Table 1 provided the study characteristics of the 22 studies included in this review, and further details are provided below: -

#### Inflammatory biomarkers

All Randomized and Case controlled trials that reported inflammatory biomarkers as an outcome were included. Two (2) studies employed the combination of aerobic and Resistance Exercises [35, 36, 61, 62]. Three (3) studies involved Resistance exercises only [44, 64, 80] while three studies utilized just aerobic exercises [59, 60, 63] Studies on Resistance exercises had two studies with Control groups that maintained their normal daily activities/habits [44, 80] and a study [64], with ‘No-exercise’ group as control. For Aerobic exercise studies; Bonato et al. [56], involved a ‘Strength-walk’ control group, Dudgeon et al. [60] had a ‘No-exercise’ control while Roos et al. [63], engaged a control group that maintained their standard clinic management. For the studies that combined both aerobic and resistant exercises; Pedro et al. [62], utilized Recreational activities as control, and Dudgeon et al. [61], maintained a ‘No-exercise’ control.

#### Cardiopulmonary function

Included studies that accessed cardiopulmonary function as an outcome were Randomized and case-control trials. Seven (7) of the studies employed the use of Aerobic exercise as intervention [66, 67, 71–75]. In addition, Terry et al. [74], included flexibility exercise. Seven (7) studies utilized Resistant exercises [36, 68–70, 73, 75, 77]. In addition, four studies [36, 68, 70, 77] included flexibility exercise. Aerobic exercise studies had control groups involved in a dietary, soft stretching and relaxation program [74], maintained current/usual level of activities during the course of the study [71, 75], did not engage in any exercise [65, 72], received counselling [66], and utilized conventional therapy [67] while Resistance exercise studies had Control groups that engaged in relaxation during the course of the study [66, 76], maintained normal/daily activities [36, 73] and did not engage in any form of exercise [68, 70, 77].

### Participants of included studies

#### Inflammatory biomarkers

A total of 406 participants (obtained at baseline) were included in this review. Participants were all HIV adults both male and female with CD4+ cell count ranging from ≤150 cell/mm^3^ to ≥1000 cells/mm^3^ with the mean age of between 18 to 60 years and were consistent on ART regimen. Three studies had participants located in Brazil [44, 62, 80], two studies were in Columbia [60, 61] while three studies were done in South Africa, USA, and New Zealand respectively [58, 63, 64].

#### Cardiopulmonary function

A total of 667 participants (obtained at baseline) were included in this review. Participants were all HIV adults both male and female with CD4+ cell count ranging from ≤200 cell/mm^3^ to ≥1000 cells/mm^3^ with the mean age of between 18 to 65 years and were consistent on ART regimen. Two studies were performed in Nigeria [66, 67], four studies in Mozambique, Ireland, India, and Rwanda respectively [65, 70, 73, 77]. Furthermore, three studies were done in the USA [36, 71, 72], three studies in Brazil [68, 74, 76] while two studies were done in Columbia and California respectively [69, 75].

#### Outcome of intervention

For the inflammatory biomarkers; three studies [44, 59, 63] reported on high sensitivity CRP, four studies reported on Interleukin-6 [60, 61, 64, 80]; three studies reported on either Interleukin-10 [44, 62, 64] and Interleukin-1β [60, 64, 80]. Also, two studies reported on Interleukin-4, 6, 10 [62, 64] or Interleukin-8 [44, 62], while one study each reported on either interleukin-5 [62], Tumor Necrosis Factor [64], Soluble Tumor Necrosis Factor-II Receptor [61], Interferon-gamma [64], Interleukin-2 [64].

For the cardiopulmonary function; six studies [36, 65, 67, 71, 73, 75] reported on maximum Oxygen uptake/Consumption (VO_2_ max), another six studies [69, 70, 72, 74, 76, 77], reported on Peak Oxygen uptake (VO_2_ peak). Smith et al. [71], and Aweto et al. [66], reported on Forced Expiratory Volume in 1 sec (FEV_1_) while WRmax was reported by Stringer et al. [75] Moreover, Vpeak, submaximal oxygen uptake (VO2 sub) and HB_5_ min were reported by Pedro et al. [76], Tidal Volume (TV) and O_2_ pulse were reported by Perna et al. [72]. Finally, a study [68] reported on the slope/intercept values for heart-Rate workload relationship.

**Table 2:** Quality appraisal /risks of bias of included studies (PEDro Tool)

Study	Sources/Potential sources of bias^a^											Score quality
	Eligibility criteria	Random allocation	Concealed allocation	Baseline similarity	Blind subject	Blind therapists	Blind accessor	Measures of key outcomes	Intention to treat	Between group	Point measure & variability	
Aweto et al. [66]	Yes	Yes	No	Yes	No	No	No	Yes	No	Yes	Yes	5/10 Mod.
Bonato et al. [59]	Yes	No	No	Yes	No	No	Yes	No	No	Yes	Yes	4/10 Low
Dolan et al. [36]	Yes	Yes	No	Yes	No	No	No	Yes	Yes	Yes	Yes	6/10 Mod.
Dudgeon et al. [61]	Yes	Yes	No	Yes	No	No	No	No	No	Yes	Yes	4/10 Low
Dudgeon et al. [60]	Yes	Yes	No	Yes	No	No	No	No	No	Yes	Yes	4/10 Low
Ezema et al. [67]	Yes	Yes	No	Yes	No	No	No	Yes	No	Yes	Yes	5/10 Mod.
Farina-tti et al. [68]	Yes	Yes	No	Yes	No	No	Yes	Yes	Yes	Yes	Yes	7/10 Mod.
Hand et al. [69]	Yes	Yes	No	Yes	No	No	Yes	No	No	Yes	Yes	5/10 Mod.
Mangona et al. [70]	Yes	Yes	No	Yes	No	No	No	Yes	No	Yes	Yes	5/10 Mod.
McDermott et al. [65]	Yes	Yes	No	Yes	No	No	No	Yes	No	Yes	Yes	5/10 Mod.
Mutimura et al. [77]	Yes	Yes	No	Yes	No	No	No	Yes	No	Yes	Yes	5/10 Mod.
Patil et al. [73]	Yes	Yes	No	Yes	Yes	No	No	No	No	Yes	Yes	5/10 Mod.
Pedro et al. [76]	Yes	Yes	Yes	Yes	Yes	No	No	No	No	Yes	Yes	6/10 Mod.
Pedro et al. [62]	Yes	Yes	Yes	Yes	No	No	No	Yes	Yes	Yes	Yes	7/10 Mod.
Perna et al. [72]	Yes	Yes	No	Yes	No	No	No	Yes	Yes	Yes	Yes	6/10 Mod.
Roos et al. [63]	Yes	Yes	Yes	Yes	No	No	Yes	Yes	Yes	Yes	Yes	8/10 High
Smith et al. [71]	Yes	Yes	No	Yes	No	No	Yes	Yes	Yes	Yes	Yes	7/10 Mod
Stringer et al. [75]	Yes	Yes	No	Yes	No	No	No	No	No	Yes	Yes	4/10 Low
Terry et al. [74]	Yes	Yes	No	Yes	No	No	No	No	No	Yes	Yes	4/10 Low
Vingren et al. [64]	Yes	Yes	No	Yes	No	No	No	Yes	No	Yes	Yes	5/10 Mod
Zanettiet al [80].	Yes	No	No	Yes	No	No	No	Yes	No	Yes	Yes	4/10 Mod
Zanettiet al [44].	Yes	No	No	Yes	No	No	Yes	Yes	Yes	Yes	No	6/10 Mod.

^a^The quality of the included studies assessed using the PEDro scale

#### Quality appraisal and risk of bias in included studies

Table 2 provided a summary of the risk of bias within included studies and further details are provided below.

**Table 3:** Data extraction of findings (except where specified, results are presented as intervention versus control group

Study	Outcome	[Int. (Mean ± SD) vs Cont (Mean ± SD); CI (…); *p* = …; d = …]
Aweto et al. [66]	Cardiopulmonary function	
	Baseline	Forced Expiratory Volume (1) (FEV1): [Int. (1.32 ± 0.62) vs Cont. (1.23 ± 0.52); *p* = 0.650]
	Post-int.	Forced Expiratory Volume (1) (FEV1): [Int. (1.98 ± 0.55) vs Cont. (1.20 ± 0.53); *p* = 0.001]
Bonato et al. [59]	Inflammatory biomarker	High sensitive C-reactive protein
	Baseline	[Walk group (1.9 ± NS) vs Walk Strength group (3.1 ± NS); *p* = NS]
	post-int.	[Walk group (0.9 ± NS) vs Walk Strength (1.8 ± NS); *p* = NS]
Dolan et al. [36]	Cardiopulmonary function	
	Baseline	VO2 max [Int. (16.9 ± 1.0) vs Cont. (15.3 ± 1.1); *p* = 0.3]
	Post-int.	Vo2 max: [int. (1.5 ± 0.8) vs Cont. (−2.5 ± 1.6); *p* < 0.001; d = NS)]
Dudgeon et al. [60]	Inflammatory biomarkers	
	Baseline	IL-6: [MOD. (3.6 ± 1.0) vs Cont. (3.6 ± 1.0); *p* = NS] IL-1β: [MOD. (5.9 ± 0.5) vs Cont. (6.2 ± 0.6); *p* = NS]
	Post-int.	IL-6: [MOD. (3.1 ± 0.6) vs Cont. (6.1 ± 2.0); *p* = NS] IL-1β: [MOD. (7.5 ± 1.7) vs Cont. (6.1 ± 0.5); *p* = NS]
Dudgeon et al. [61]	Inflammatory biomarkers	
	Change after intervention	IL-6 [(2.7 ± 0.27);*p* = NS] IL-1β: [(3.8 ± 0.07); *p* = NS]
		sTNFrII: [(2.8 ± 1.94); *p* = 0.05 at 30-mins for LOW group and *p* = 0.05 at MOD group]
Ezema et al. [67]	Cardiopulmonary function	
	Baseline	VO2 max [Int. (23.00 ± 2.54) vs Cont. (24.00 ± 2.54); *p* = 0.878]
	Post-int.	VO2 max: [int. (30.87 ± 4.37) vs Cont. (23.87 ± 2.65); *p* < 0.000; d = NS)]
Farinatti et al. [68]	Cardiopulmonary function	
	Baseline	Slope for heart rate-workload relationship α [Int. (1.47 ± 0.4) vs Cont. (1.5 ± 0.3); *p* = NS]
		Slope for heart rate-workload relationship α [Int. (1.18 ± 0.2) vs Cont. (1.67 ± 0.5); *p* = NS
	After	Intercept for heart rate-workload relationship*bpm* [Int. (76.5 ± 9.4) vs Cont. (78.5 ± 8.9); *p* = NS]
		Intercept for heart rate-workload relationship*bpm* [Int. (67.4 ± 8.2) vs Cont. (76.4 ± 7.0); *p* = NS]
		For slope and Intercept, *p* < 0.05)
Hand et al. [69]	Cardiopulmonary function	
	Baseline	VO2 peak [EX. (31.6 ± 2.1) vs Cont. (29.4 ± 0.3); *p* = NS]
	After	VO2 peak [EX. (39.9 ± 1.9) vs Cont. (29.4 ± 2.7); *p* < 0.01]
Mangona et al. [70]	Cardiopulmonary function	
	Baseline	VO2 peak [FEG (33.2 ± 5.5) vs Cont. (33.0 ± 5.5); *p* = NS]
		VO2 peak [PEG. (31.3 ± 9.1) vs Cont. (33.0 ± 5.5); *p* = NS]
	After	VO2 peak [FEG] (31.8 ± 7.1) vs Cont. (33.2 ± 3.9); *P* = 0.008]
		VO2 peak [PEG. (34.8 ± 9.9) vs Cont. (33.2 ± 3.9); *p* = 0.010]
Mcdermott et al. [65]	Cardiopulmonary function	
	Baseline	VO2 max [Int. (33.2 ± 9.6) vs Cont. (36.7 ± 8.6.); *p* = 0.24]
	Post-int.	Vo2 max [int. (37.4 ± 8.9) vs Cont. (39.0 ± 9.6);*p* = 0.85; d = NS)]
Mutimura et al. [77]	Cardiopulmonary function	
	Change comparison between the groups	VO2 peak [(4.7+ 0.56); *p* = < 0.0001)
Patil et al. [73]	Cardiopulmonary function	
	Baseline	VO2 max [Int. (31.37 ± 2) vs Cont. (29.5 ± 2); *p* = NS]
	Post-int.	Vo2 max: [int. (33.27 ± 2) vs Cont. (29.6 ± 2); *p* = 0.001; d = NS)]
Pedro et al. [76]	Cardiopulmonary function	
	Baseline	VO2 peak [Int. (26.3 ± 4.7) vs Cont. (26.3 ± 8.9); *p* = 0.0001]
		VO2 sub [Int. (12.6 ± 2.8) vs Cont. (12.7 ± 2.9); *p* = NS]
		Vpeak [Int. (7.2 ± 0.9) vs Cont. (6.8 ± 1.5); *p* = NS]
		HR5min[Int.116 ± 21) vs Cont. (118 ± 17); *p* = NS]
	Post-int.	VO2 peak: [int. (31.6 ± 5.6) vs Cont. (27.6 ± 7.6); p ≤ 0.05; ES = 0.55d = NS)]
		VO2 sub [Int. (13.7 ± 3.1) vs Cont. (15.6 ± 3.1); *p* = NS; ES = − 0.57, d = NS]
		Vpeak [Int. (8.4 ± 1.2) vs Cont. (7.0 ± 1.7); *p* ≤ 0.05; ES = 0.81]
		HR5min[Int.105 ± 21) vs Cont. (113 ± 14); p ≤ 0.05; ES = − 0.43]
Pedro et al. [62]	Inflammatory biomarkers	
	Baseline	IL-4[Int. (11.4 ± 5.1) vs Cont. (11.0 ± 5.1); *p* < 0.05]
		IL-5 [Int. (1.4 ± 0.4) vs Cont. (1.6 ± 0.9); *p* < 0.05]
		IL-10 [Int.(7.3 ± 0.6) vs Cont. (7.3 ± 1.0); *p* < 0.05]
		IL-8[Int.8.0 ± 4.4) vs Cont. (7.1 ± 5.1); *p* < 0.05]
	Post-int.	IL-4[Int. (9.7 ± 6.3) vs Cont. (13.8 ± 8.3); *p* > 0.05; ES = − 0.60]
		IL-5 [Int. (1.3 ± 0.6) vs Cont. (2.4 ± 2.1); *p* > 0.05; ES = − 0.68]
		IL-10 [Int.(7.1 ± 0.4) vs Cont. (7.3 ± 1.0); *p* > 0.05; ES = − 0.79
		IL-8[Int.5.4 ± 2.3) vs Cont. (8.1 ± 6.0); *p* < 0.03; ES = − 0.87]
Perna et al. [72]	Cardiopulmonary function	
	Baseline	VO2 peak: [Compliant (28.7 ± 7.5) vs control (28.3 ± 9.6); *p* = NS]
		VO2 peak: [Compliant (28.7 ± 7.5) vs non-compliant (25.4 ± 9.1); *p* = NS]
		O_2_ pulse [Compliant (11.7 ± 3.4) vs control (12.3 ± 3.2); *p* = NS]
		O_2_ pulse [Compliant (11.7 ± 3.4) vs non-compliant (13.0 ± 2.6); *p* = NS]
		Tidal Volume [Compliant (2.0 ± 0.6) vs control (12.3 ± 3.2); *p* = NS]
		Tidal Volume [Compliant (2.0 ± 0.6) vs non-compliant (13.0 ± 2.6); *p* = NS]
	Post-int.	VO2 peak: [Compliant (32.2 ± 7.2) vs control (26.5 ± 8.4); *p* < 0.01]
		VO2 peak: [Compliant (32.2 ± 7.2) vs non-compliant (25.4 ± 8.8); *p* = NS]
		O_2_ pulse [Compliant (13.0 ± 3.1) vs control (11.4 ± 2.8); *p* < 0.01]
		O_2_ pulse [Compliant (13.0 ± 3.1) vs non-compliant (13.4 ± 2.5); *p* = NS]
		Tidal Volume [Compliant (2.2 ± 0.6) vs control (2.0 ± 0.7); *p* < 0.01]
		Tidal Volume [Compliant (2.2 ± 0.6) vs non-compliant (2.0 ± 0.7); *p* = NS]
Roos et al. [63]	Inflammatory biomarkers	
	Baseline	hs-CRP [Int. (8.58 ± 1.29) vs Cont. (5.45 ± 1.02); *p* ≥ 0.05]
	At 6 months	hs-CRP [Int. (− 0.43 ± 1.04) vs Cont. (0.57 ± 0.60); *p* = 0.08]
	At 12 months	hs-CRP [Int. (0.31 ± 1.29) vs Cont. (0.21 ± 0.59); *p* = 0.08;d = NS]
Terry et al. [74]	Cardiopulmonary function	
	Baseline	VO2 peak [Diet/Exer (32 ± 5) vs Diet only (34 ± 7); *p* > 0.05]
	After	VO2 peak [Diet/Exer (40 ± 5) vs Diet only (35 ± 8); *p* = 0.001]
Smith et al. [71]	Cardiopulmonary function	
	Baseline	FEV_1_ [Int. (4.0 ± 0.6) vs Cont. (3.6 ± 0.9); *p* = NS]
		VO2 max [Int. (34.9 ± 5.7) vs Cont. (31.0 ± 55.9); *p* = NS]
	After	FEV_1_[Int (4.1 ± 0.8) vs Cont. (31.0 ± 5.9); *P* = 0.32]
		VO2 max [Int. (37.5 ± 6.1) vs Cont. (32.0 ± 6.9); *p* = 0.09]
Stringer et al. [75]	Cardiopulmonary function	
	%, pre – post	WR max [MOD (179 ± 21) vs Cont. (166 ± 29); *p* < 0.01]
		WR max [HEAVY. (162 ± 10) vs Cont. (166 ± 29); *p* < 0.01]
		VO2 max [MOD 2.2 ± 0.2) vs Cont. (2.0 ± 0.3); *P* = 0.32]
		VO2 max [HEAVY (1.9 ± 0.1) vs Cont. (2.0 ± 0.3); *p* = 0.01]
Vingren et al. [64]	Inflammatory biomarkers	
	Baseline	WR max [MOD (4.9 ± 3) vs Cont. (− 9 ± 5); *p* < 0.01]
		WR max [HEAVY. (21 ± 15) vs Cont. (− 9 ± 5); *p* < 0.01]
		VO2 max [MOD (− 0.1 ± 0.1) vs Cont. (− 0.2 ± 0.1); *P* = 0.32]
		VO2 max [HEAVY (0.2 ± 0.1) vs Cont. (− 0.2 ± 0.1); *p* = 0.01
		IL-1β [MOD. (0.2 ± 0.1) vs Cont. (0.3 ± 0.4); *p* = NS]
		IL-2 [MOD. (2.6 ± 1.7) vs Cont. (4.8 ± 8.1); *p* = NS]
		IL-4 [MOD.(16.0 ± 12.3) vs Cont. (17.7 ± 15.8); *p* = NS]
		IL-6[MOD.(3.4 ± 1.3) vs Cont. (2.4 ± 0.9); *p* = NS]
		IL-10 [MOD.(114.9 ± 64.8) vs Cont. (59.3 ± 16.0); *p* = NS]
		IFN-ϒ[MOD.(14.2 ± 16.1) vs Cont. (10.5 ± 8.8); *p* = NS]
		TNF-α [MOD.(16.4 ± 9.6) vs Cont. (10.3 ± 5.4); *p* = NS]
	Post-int.	IL-1β[MOD. (2.6 ± 1.7) vs Cont. (4.8 ± 8.1); *p* = NS]
		IL-2 [MOD. (2.4 ± 2.0) vs Cont. (4.6 ± 7.6); *p* = NS]
		IL-4 [MOD.(14.0 ± 10.0) vs Cont. (17.7 ± 15.8); *p* = NS]
		IL-6[MOD.(3.9 ± 1.8) vs Cont. (2.7 ± 1.3); *p* = NS]
		IL-10 [MOD.(108.5 ± 67.0) vs Cont. (58.2 ± 17.8); *p* = NS]
		IFN-ϒ[MOD.(11.4 ± 2.2) vs Cont. (10.3 ± 8.8); *p* = NS]
		TNF-α [MOD.(15.4 ± 8.0) vs Cont. (9.9 ± 3.7); *p* = NS]
Zanetti et al. [44]	Inflammatory biomarkers	
	(pre- post)	IL-1β[NLRT (−1.1 ± 1.6) vs Cont. (0.19 ± 0.44); *p* < 0.0001]
		IL-6 [NLRT (− 2.3 ± 0.4) vs Cont. (0.09 ± 0.3); *p* < 0.0001]
		IL-8[NLRT (− 5.5 ± 3.4) vs Cont. (− 0.15 ± 2.3); *p* < 0.0001]
		IL-10[NLRT (1 ± 0.4) vs Cont.(− 0.05 ± 1.3); *p* < 0.0001]
		TNF-α [NLRT (− 5.72 ± 2.7) vs Cont.(1.33 ± 0.3); *p* < 0.0001]
Zanetti et al. [8]	Inflammatory biomarkers	
	(pre- post)	CRP [(mean + S.D) = NS; *p* = (*p* = .005)]

#### Eligibility criteria

Authors from the twenty-two (22) studies reported on the Inclusion and Exclusion criteria they used in recruiting and screening participants for their respective studies. Hence the low risk of bias was evident in the whole studies.

#### Random allocation

Twenty-one studies (21) reported on using randomization process to allocate their eligible participants to the different groups (Experimental: low, moderate or heavy, and Control group) while six out of them did not give a description of the method used for the randomization [44, 64, 68, 71, 72, 80]. One study [58] performed a pretest-posttest clinical trial. Low risk for selection bias was evident for the selected studies because they complied with randomisation in drawing the study samples.

#### Concealment of allocation

This is an overall 70% of selection bias as only seven studies [60–63, 66, 67, 77] out of the 23 studies provided information on how concealment was used in allocating the participants the into the different study groups.

#### Baseline similarity

All the studies included participants who had similar baseline characteristics of measured variables. Low risk of selection bias is evident in the studies.

#### Bias on blinding

For the blinding of subjects and therapists during the intervention period, two studies [36, 48, 62, 73] reported on performing a single-blinded trial. For the blinding of those evaluating the outcome variables, five studies [59, 63, 68, 69, 71] have a low risk of bias here by reporting on the blinding of the assessors/evaluators that determined the measurement of the outcome variables.

#### The bias of outcome measurement from < 85% of initial participants (incomplete outcome data)

Form the total (1073) number of subjects randomized into different groups, 268 individuals dropped out from the respective studies accounting for ~ 25% of withdrawal rate ranging from 5% [36] to 39.12% [63]. Hence, an outcome measure bias exists as 14 out of the 22 selected studies carried out the post-intervention assessment of the key variables being studied using < 85% of subjects that were initially randomized into groups while one study did not report on the incomplete outcome or drop-outs [80]. Eight studies have a low risk of incomplete outcome bias by having a retention rate ranging from 85% [65, 70] to complete outcome [44, 64, 68] or complete in-patient; included the non-compliance subjects in the final outcome assessment [70, 71] and carrying out an intention-to-treat analysis for the drop-outs [36, 62, 63]. The compliance rate was also mentioned by almost all the studies.

#### Effects of intervention

Except where otherwise stated, the effects of the intervention are reported as a comparison of the intervention versus the control group.

### Inflammatory biomarkers

#### High sensitivity C-reactive protein

Three studies provided data on high sensitivity CRP. One good quality trial with a score of six out of ten points [62] reported no statistically significant change (*p* = 0.08) in mean hs-CRP between groups while one fair and another good quality trials with a score of four out of ten points, respectively [44, 59], reported a significant reduction in hs-CRP level (*p* = 0.004 and *p* = 0.012, respectively) between the groups.

#### Interleukin-6

Four studies gave details on pro-inflammatory Interleukin-6. One fair-quality trial with a score of four out of ten points [44] reported on a statistically significant (*p* = .005) decline while another good quality trial with a score of five out of ten points [64] and a fair quality trial with a score of four out of ten points [60] stated that there was no significant change (*p* = 0.60) in the level of IL-6 between the groups. Dudgeon et al. [61], another fair-quality study with a score of four out of ten points, reported no significant change in all the cytokine levels, including IL-6 (*p* = 06) post 30 and 60-mins in the MOD group for each individual exercise session.

#### Interleukin-10

Three studies reported on anti-inflammatory Interleukin-10. A fair-quality trial with a score of four out of ten points [44] reported on a significant increase IL-10 levels (*p* = .030) in IL-10 between the groups while one good quality study with a score of seven out of ten points, respectively [62] and another fair-quality study [64] with a score of five out of ten points stated that no statistically significant change (*p* > 0.05) exists between the groups. Some studies that assessed for pro-inflammatory Interleukin-1β [60, 64] concluded that there was no significant effect (*p* > 0.05) of exercise training on IL-1β while a good quality RCT [80] with a score of six out of ten points reported a statistically significant decrease (*p* = 0.004) in CRP, following the intervention.

#### Interleukin-8, Interleukin-5, interferon-gamma, Interleukin-2, tumor necrosis factor and soluble tumor necrosis factor-II receptor

One good quality RCT on Interleukin-8 [62] with a score of six out of ten points stated that there was no statistical difference (*p* = 0.03) existing between groups while another study [44], reported a significant reduction (*p* = .010) in the biomarker. Vingren et al. [64], reported no significant effect (*p* > 0.05) of the intervention on the biomarkers - Tumor Necrosis Factor, Interferon-gamma, Interleukin-2. Dudgeon et al. [61], concluded that there was no significant decrease (*p* = 0.6) in Soluble Tumor Necrosis Factor-II Receptor while Pedro et al. [62], reported no statistically significant difference (*p* > 0.05) on interleukin-5, 4, 10.

Five quality studies reported a significant change in inflammatory cytokines in the exercise group following the period of the intervention in relation to the control group while three studies reported no significant effect (*p* > 0.05) of the intervention on the biomarkers between the exercise and control group. An analysis of cardiopulmonary function outcomes is presented in Table 3.

**Table 1:** Study characteristics

Study (Country), Design, Attrition	Participants	Interventions	Outcomes	Conclusion
Aweto et al. [66] (Nigeria), RCT, 17.5%	People living with HIV on HAART (*n* = 40).	Intervention: Aerobic exercise training three times a week for 6 weeks and counseling. Control: Control group received Counseling.	Cardiopulmonary function: FEV_1_ assessed at baseline and at 6th week.	FEV_1_ significantly improved in the study group compared with the control group
Bonato et al. [59] (New Zealand), Pilot clinical trial, 28.6%	Sedentary HIV-infected persons (cART-treated) with metabolic complications (*n* = 49).	Intervention: 12-week exercise training, consisting of three sessions per week of 60 min brisk walking with (strength-walk group) or without (walk group) 30 min circuit-training. Control: Pre-test- post-test walk-strength group.	Inflammatory biomarkers: High sensitivity CRP, Interleukin-6, 18, D-dimer, soluble CD14. Outcome assessed at baseline and at 12 weeks.	Brisk walking, with or without strength exercise, could improve lipid profile and inflammatory markers in chronic HIV infection.
Dolan et al. [36] (USA)^,^ RCT, 5%	HIV-infected women with increased waist-hip ratio and self-reported fat redistribution (*n* = 34).	Intervention: Home based aerobic exercise (60–75% of MaxHR, 20–30 min) and progressive resistant exercise with equipments (60–80% of 1-RM, 3–4 sets of 8–10 RM). Control: Maintained normal activities.	Cardiopulmonary function: VO_2max._ Outcome assessed at baseline and at 16th week.	There was a significant improvement in VO_2max_ after 16 weeks of aerobic training relative to the control group.
Dudgeon et al. [61] (Columbia), RCT, 21.63%	HIV infected men (*n* = 37).	Intervention: For MOD group, 30 mins of aerobic exercise at 60–65% of age-predicted max HR and upper-body/lower-body resistant exercise (60% of 1-RM). For LOW group, 60 min of low intensity exercise (50% of age predicted max HR). Control: Did not receive any activity.	Inflammatory biomarkers: interleukin-6, soluble TNfrereceptor II. Outcome assessed at baseline 30, 30-mins post exercise, 60-mins post exercise.	There was an increase in Il-6 from baseline to post 30 (31%) and post 60 (23%) in the MOD group while the LOW group had a 3.5% decrease in sTNFrII (*p* < 0.05) at 30-mins post exercise compared with baseline.
Dudgeon et al. [60] (Columbia), RCT, 31.54%	HIV infected men (*n* = 111).	Intervention: 30 mins of moderate intensity aerobic exercise training on a treadmill or stationary cycle (60–75% of age-predicted max HR) and lower-body/upper-body resistant exercise (12-RM). Control: Did not receive any activity	Inflammatory biomarkers: Interleukin-6, 1β. Outcome accessed at baseline and 6th week	Although, there was no detectable change in the level of IL-6, IL-1β was significantly elevated.
Ezema et al. [67] (Nigeria), RCT, 9.1%	People Living with HIV who are receiving ART (*n* = 30).	Intervention: Moderate intensity continuous aerobic exercise training (60–79% of the maxHR, 40–60 min, 3 times/week) on a treadmill. Control: Conventional therapy involving ART and counseling.	Cardiopulmonary function: VO_2max._ Outcome assessed at baseline and at 8th week.	Moderate intensity continuous exercise program had a significant effect on VO_2max_.
Farinatti et al. [68] (Brazil), RCT, nil	Seropositive patient treated with HAART.	Intervention: Aerobic exercise on cycle ergometer (30 mins); strengthening exercises (3 sets of 12 reps; Flexibility exercise (10 mins). Control: Participants did not receive any exercise.	Cardiopulmonary function: VO_2max._ Outcome assessed at baseline and at 12th week.	Overall training can improve aerobic fitness of HIV-infected patients with no negative effect on their immunological function.
Hand et al. [69] (Columbia), RCT, 34.88%	HIV-infected men and women (*n* = 43).	Intervention: Aerobic exercise training on threadmill (30 mins, 50–70% of age predicted MHR) and upper-body/lower body resistance training (20 mins, 12-RM). Control: wait-list	Cardiopulmonary function: VO2 max. Outcome assessed at baseline and at 6^th^week.	There was a significant increase in estimated VO2 max (*p* = 0.001) using moderate exercise training
Mangona et al. [70] (Mozambique)^,^ RCT, 15%	HIV+ African Women taking ART (*n* = 53).	FEG: 20 mins of cycling at 60–85% of V_2_ peak and muscular endurance; circuit training consisting of 6 free weight exercises (15-RM); stretching exercises (*n* = 19) PEG: recreational activities Control: No exercise	Cardiopulmonary function: VO2 max. Outcome assessed at baseline and at 12th week.	Cardiopulmonary fitness increases significantly in VO2 peak (FEG: 14.8%; PEG: 11.1%) with no significant difference in the CG
McDermott et al. [65] (Ireland)^,^ RCT, 15.4%	HIV+ patients without any known cognitive function (*n* = 13).	Supervised session of exercise training (ergometer, treadmill, cross trainer); 40–75% of HRreserve and unsupervised session (jogging, brisk walking, cycling) 3 times per week. Control: Advised to continue with normal routine.	Cardiopulmonary function: VO_2max._ Outcome assessed at baseline and at 16th week.	Aerobic exercise had no effect on aerobic fitness or cognitive function.
Mutimura et al. [77] (Rwanda), RCT, 4%	HIV+ patients with moderate to severe Body fat redistribution (*n* = 150).	Supervised training program (stretching, aerobic and strengthening exercises) 45–75% of age predicted Max HR. Control: Participants did not perform any exercise training.	Cardiopulmonary function: VO_2peak._ Outcome assessed at baseline and at 6th month.	Exercise training positively improved cardiorespiratory fitness in HAART-treated HIV+ Africans.
Patil et al. [73] (India), RCT, 40%	HIV-positive females (*n* = 40).	Aerobic exercise (brisk walking, 50–70% of VO_2max_) and resistance exercise via free weight. Control: Advised to continue their routine level of daily tasks and activities.	Cardiopulmonary function: VO_2peak._ Outcome assessed at baseline and at 8th week.	Moderate intensity improved aerobic capacity in experimental HIIV group.
Perna et al. [72] (South Florida), RCT, 34.88%	Symptomatic HIV-1 seropositive men and women (*n* = 43).	An interval cycling exercise program (3 times per week) for 3 months; 45 mins, 70–80% of mHR. Control: Continued with usual activity (wait-list).	Cardiopulmonary function: VO_2peak_, O_2_ pulse, Tidal volume. Outcome assessed at baseline and at 12th week.	Functional aerobic limitations common in HIV-infected individuals can be reversible through exercise adherence.
Pedro et al. [76] (Brazil), RCT, 43.1%	Adults living with HIV (*n* = 58).	Intervention: Concurrent training (15–20 min of aerobic exercise; 50–70% of HRrest plus 40 mins of resistant exercise; 2–3 sets of 8–12 RM) 3 times per week The resistant exercise involves free weights & machine. Control: Optional Recreational activities (once/twice per week).	Cardiopulmonary function: VO_2peak_, VO_2sub_, peak speed. Outcome assessed at baseline and at 16th week.	Concurrent training was effective in improving cardiopulmonary fitness and endurance.
Pedro et al. [62] (Brazil), RCT, 42.86%	Adults living with HIV (*n* = 49).	Concurrent training consisting of 20-min aerobic exercise training on a treadmill (50–70% of HR) and resistant training (8 exercises via free weights or machine, 2–3 sets of 8–12 RM). Control: 60 min of recreational activities comprising of dancing, walking, stretching.	Inflammatory biomarkers: Intrleukin-4, 5, 6, 8, 10, Tumor Necrosis Factor-alpha, IFN-ϒ. Outcome accessed at baseline and 16th week.	Concurrent training decreased the pro-inflammatory effects of IL-5, IL-5, 8, 10 in HIV infected people undergoing ART.
Smith et al. [71] (USA), RCT, 18%	HIV-1 Infected adults (*n* = 60).	Intervention: Supervised aerobic exercise training program (3x per wk) for 30 mins, 60–80% of VO2 max. Control: Continued with usual activity (wait-list)	Cardiopulmonary function: FEV1, Vo2 max. Outcome assessed at baseline and at 12th week.	There was a beneficial increase in VO_2max_ by 2.6 mL/kg.
Stringer et al. [75] (California), RCT, 23%	HIV positive subjects (*n* = 34).	Intervention: For MOD, aerobic exercise (80% of LAT work rate, 3 times per week). For HEAVY: aerobic exercise (50% of difference between their LAT and their VO2 max) for 30–40 min on cycle ergometer. Control: Maintained current level of activity without change.	Cardiopulmonary function: FEV1, VO_2max_. Outcome assessed at baseline and at 6th week.	Aerobic fitness increased was significantly in both the EX groups relative to the control group.
Roos et al. [63] (South Africa), RCT, 39.2%	HIV-infected individuals with the risk factor of Ischemic Heart Diseases (*n* = 84).	Intervention: Education and home-based pedometer walking program to improve participants’ activity.30 min walking program 3 or 5 times a week over a 12-week period. Control: continued with standard clinic management	High Sensitivity CRP. Outcome assessed at baseline and at 12 weeks.	Intervention had no effect on CRP.
Terry et al. [74] (Brazil), RCT, 28.57%	Carriers of HIV-1 virus who had hyperlipidemia (*n* = 42).	Intervention: Aerobic exercise training (30 mins of the target intensity) using treadmill and stretching exercises. Control: 45 mins of soft stretching and relaxation routine without significant elevation of HR	Cardiopulmonary function: VO_2max_. Outcome assessed at baseline and at 12th week.	Intervention resulted in a significant improvement in VO_2max_ for the Diet/EX group compared to the Diet-only group.
Vingren et al. [64] (USA), RCT, nil	Men infected with HIV and recently admitted to an inpatient substance facility (*n* = 30).	Intervention: Progressive overload Resistance training program comprising of free weight and cable controlled exercise (3x per week, 3–5 sets of 5–12 reps). Control: Maintained usual daily activities.	Inflammatory biomarkers: IL-10, 6, 4, 2, 1β, IFN-ϒ Outcome assessed at baseline and at 6th week.	Intervention had no effect on basal concentration of circulating cytokines for men living with HIV and undergoing treatment for substance abuse.
Zanetti et al. [80] (Brazil), RCT, nil	Previously sedentary people infected with HIV (*n* = 30).	Intervention: Non-linear Resistance intervention with free weight exercises on 3 alternate days. Control: Maintained daily habits.	Inflammatory biomarkers: Interlleukin-6, 8, 10, 1β. Outcome assessed at baseline and at 12th week.	There was an increase in IL-10, and a decrease in IL-1β, IL-6, IL-8, TNF-α.
Zanetti et al. [44] (Brazil)^,^ RCT, nil	People living with HIV that are on HAART (*n* = 30).	Intervention: Supervised nonlinear Resistance training program 3times per week on nonconsecutive days via free weight. Control: Maintained usual daily activities	High Sensitivity CRP. Outcome assessed at baseline and at 12th week.	There was a significant reduction in CRP levels in inflammatory markers in PLHIV.

#### Cardiopulmonary function

Three studies [67, 73, 75], reported a significant improvement (*p* = 0.0001; *p* = 0.001; *p* = < 0.01) in aerobic fitness/capacity in the study group. Eight studies [36, 67, 69, 70, 72, 74, 76, 77] reported statistically significant (*p* < 0.05) improvement in the peak/maximum oxygen uptake post-training period in the intervention group while two studies [65, 66] reported no significant (*p* > 0.05) improvement of the intervention on the stated outcomes. Two other trials [66, 70] reported a significant increase (*p* < 0.01) effect, respectively, in the mean of the FEV_1_ in the study group, compared to that the control group. Farinatti et al. [68], observed a significant improvement (*p* < 0.05) in the slope/intercept values for heart-Rate workload relationship. Hence, a total of 13 studies reported a statistically significant improvement in the cardiopulmonary-related parameters while two other studies reported no significant improvement in the study group (Table 3).

#### Meta-analyses- effect of interventions

Based on the observed homogeneity of intervention effects, this review performed three meta-analyses across three subgroup comparisons, which included meta-analyses of various exercise interventions on cardiopulmonary function - V0_2_ Max and inflammatory biomarkers (IL-6 and IL-1β). The sub-group comparisons of the meta-analyses were;

1. Aerobic exercises plus resistance exercises compared with normal activities/wait list as a control.
2. Aerobic exercises only compared with normal activities/wait list as a control.
3. Resistance exercises only compared with normal activities/wait list as a control.
4. Physical exercises (i.e. all types of exercises) compared with normal activities/wait list as a control.

For the VO_2_ Max outcome, five studies [36, 69, 70, 73, 76] compared aerobic exercises plus resistance exercises group with normal activities/wait list control group. Five studies [65, 67, 71, 72, 74] compared aerobic exercises only with normal activities/wait list control group.

For IL-6 outcome, two studies [60, 61] compared aerobic exercises plus resistance exercises group with normal activities/wait list control group. Also, two studies [64, 80] compared resistance exercises only with normal activities/wait list control group.

For IL-1β outcome, one study [60] compared aerobic exercises plus resistance exercises group with normal activities/wait list control group. However, two studies [64, 80] compared resistance exercises group with normal activities/wait list control group. Therefore, ‘physical exercises’ was considered as a sub-group to include the three studies in the meta-analyses.

#### Heterogeneity test

Systematic heterogeneity (*p* < 0.1) was observed in the 3 meta-analyses conducted. This could be as a result of variances in gender, types of intervention, duration of the intervention and study location. Therefore, a random effect model was used for the meta-analyses.

### Results of Meta-analyses

#### Cardiopulmonary function (VO_2_ max)

Twelve of the twenty-two included studies (50%) assessed VO_2_ Max/peak as a cardiopulmonary function outcome. However, only ten [36, 65, 67, 69–74, 76] of the studies were included in meta-analyses, while the other two studies [75, 77] were excluded due to incomplete data outcome reporting and failure to send the data after 3 consecutive emails.

**Fig. 2:**
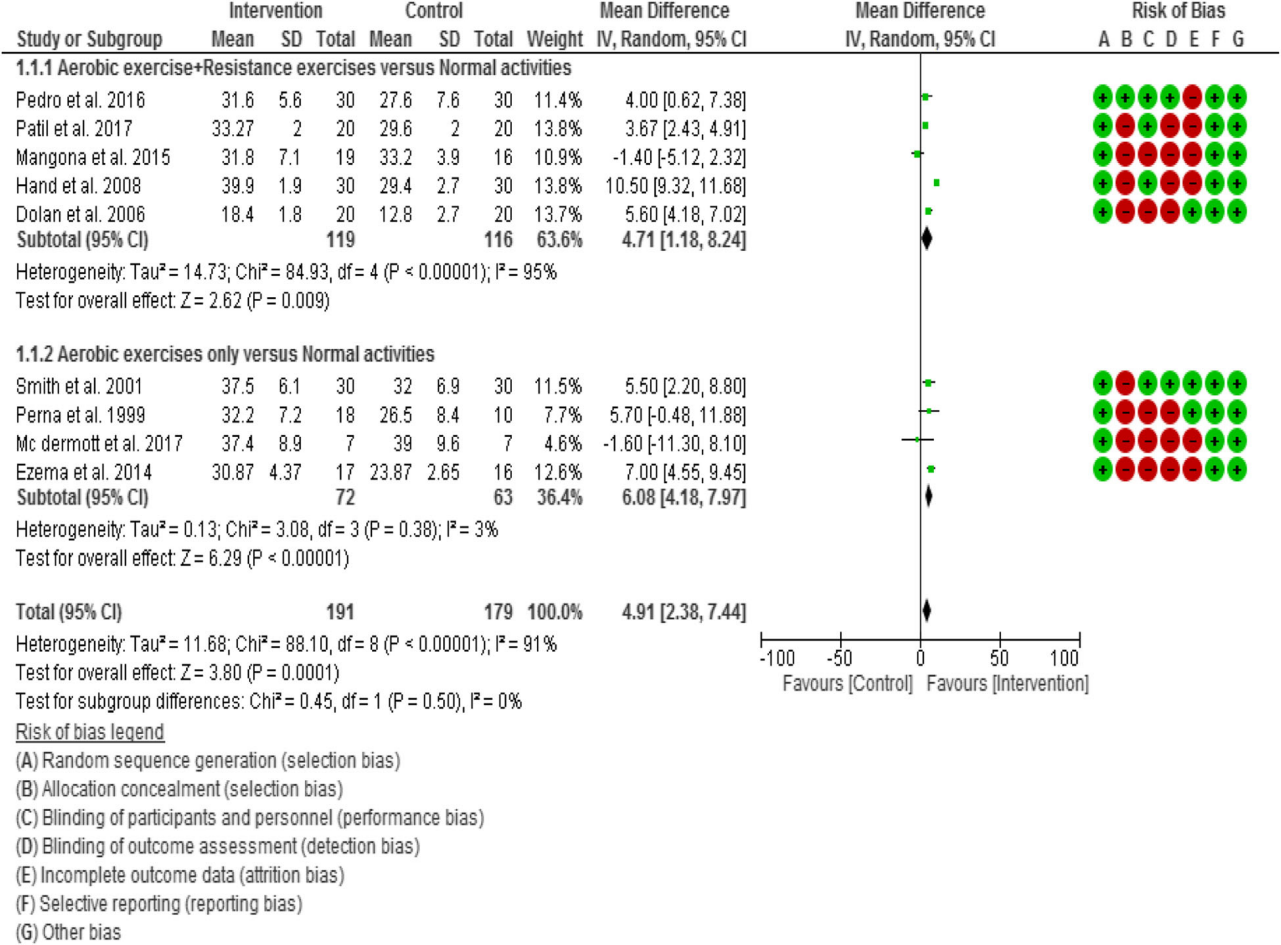
Forest plot for VO_2_ Max

There was an overall statistically significant (Z = 3.80, *p* < 0.0001) change in VO_2_ Max between comparison groups. Results demonstrated a statistically significant (Z = 2.62, *p* = 0.009) trend towards an increase in V0_2_ Max in subjects in the aerobic exercises plus resistance exercises group compared with normal activities control group (i.e. in favour of the intervention). There was also a statistically significant (Z = 6.29, *p* < 0.0001) trend towards an increase in VO_2_ Max in subjects in the aerobic exercises group compared with normal activities control group (i.e. in favour of the intervention) (Fig. 2).

#### Interleukin-6 (IL-6)

**Fig. 3:**
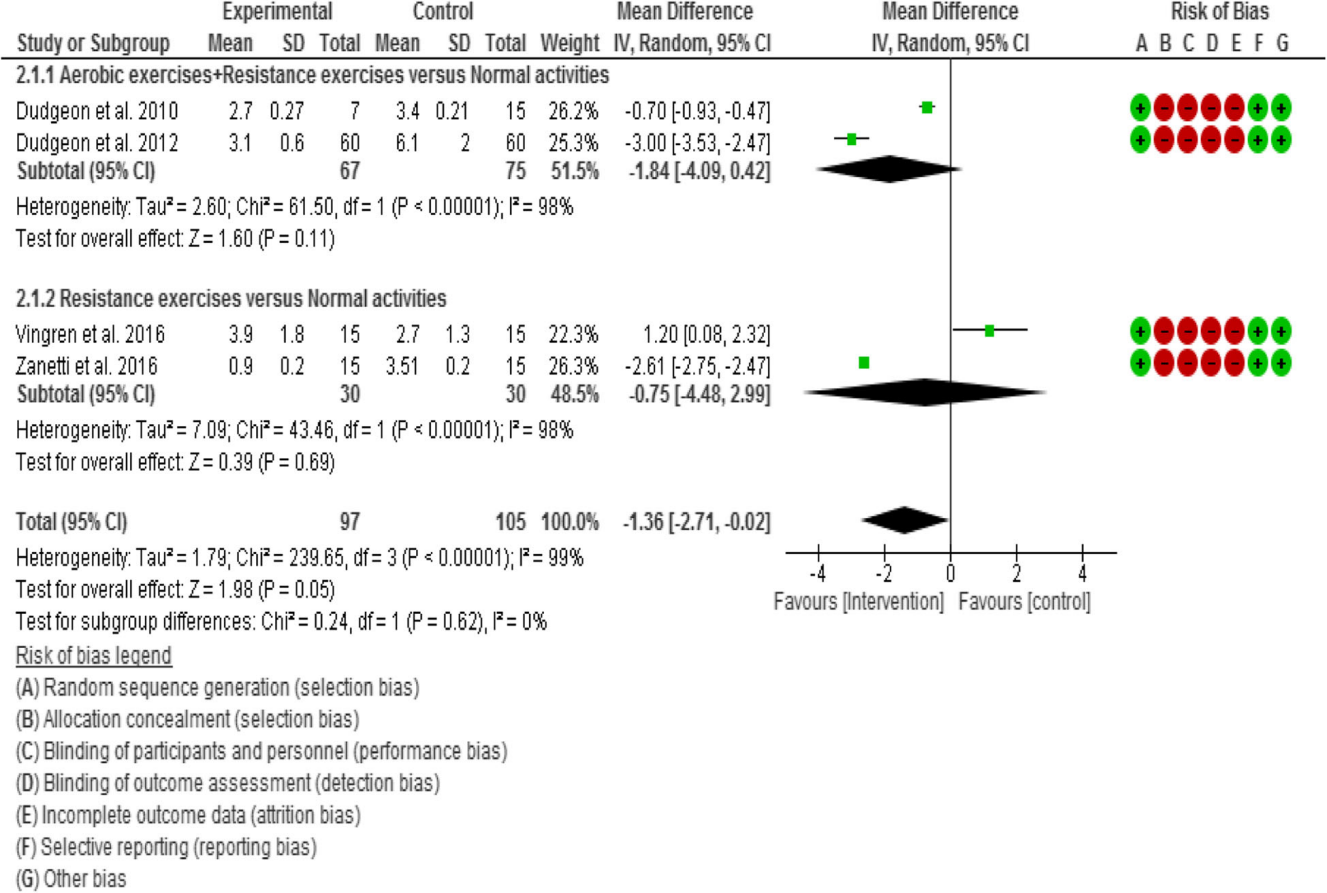
Forest plot for IL-6

Four of the twenty-two included studies (17.39%) evaluated the effect of exercises on IL-6. The four studies [44, 60, 61, 64] were included in the meta-analyses. There was no overall statistically significant (Z = 1.98, *p* = 0.05) change in IL-6 level between comparison groups. Results demonstrated no statistically significant (Z = 1.60, *p* = 0.11) trend towards a decrease in IL-6 in subjects in the aerobic exercises plus resistance exercises group compared with normal activities control group (i.e. in favour of the intervention). There was also no statistically significant (Z = 0.39, *p* = 0.69) trend towards a decrease in IL-6 in subjects in the resistance exercises group compared with normal activities control group (i.e. in favour of the intervention) (Fig. 3).

#### Interleukin-1β

**Fig. 4:**
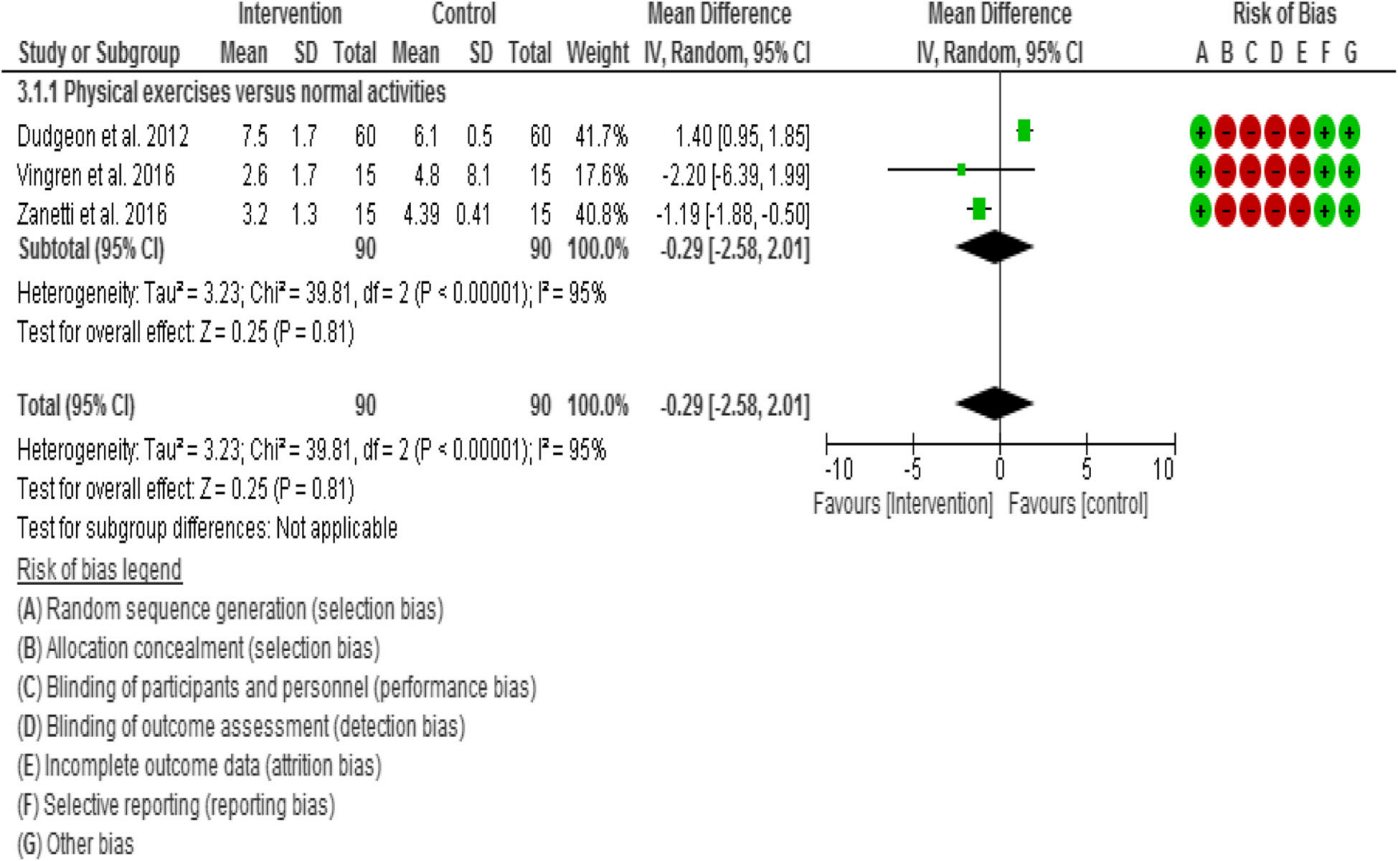
Forest plot for IL-1β

Four of the twenty-two included studies (17.39%) evaluated the effect of exercises on IL-1β. However, three studies [44, 60, 64] were included in the meta-analyses, while one study [61] was excluded due to incomplete data outcome reporting and failure to send the data after 3 consecutive emails. Results demonstrated no statistically significant (Z = 0.25, *p* = 0.81) trend towards a decrease in IL-1β in subjects in the physical exercises group compared with normal activities control group (i.e. in favour of the intervention) (Fig. 4).

## Discussion

Twenty-two trials determining the efficacy of various exercise interventions for improving the level of inflammatory biomarkers and cardiopulmonary functioning in patients living with HIV infection undergoing ART were reviewed. Studies included for the review were mainly of fair methodological quality with a moderate risk of bias. Five studies stated that there exists a significant change in inflammatory cytokines in the exercise (EX) group following the period of the intervention in relation to the control (CON) group while three studies reported no significant effect of the intervention on the biomarkers between the EX and CON group. Evidence from 12 studies reported a statistically significant improvement in the cardiopulmonary related parameters while two other studies reported no significant improvement in the study.

Meta-analyses suggest that performing constant or interval aerobic exercise or a combination of constant aerobic exercise and progressive resistive exercise for at least 15 min, 23 times per week for at least 4 weeks may improve the cardiopulmonary functioning (maximum/peak oxygen consumption) of the individual. Results suggest that physical exercise is safe for medically stable adults living with HIV. This result is supported in that few or no reports of adverse effects of exercise as an intervention among studies as well as the absence of change in CD_4_^+^ cell count and viral load in the subjects. It is also based on participants who completed the exercise interventions and for whom there were adequate follow-up data which was clearly stated. Twenty-two studies were incorporated into the update of this systematic review, out of which 14 of them were included in the meta-analyses [36, 44, 60, 61, 65, 67, 69–74, 76, 80].

For the studies that assessed inflammatory biomarkers: four out of eight [44, 60, 61, 64] studies were included in the meta-analyses conducted for interleukin-6 and interleukin-1β. The meta-analyses revealed that exercises had no significant effects on the plasma level of these inflammatory biomarkers compared to the control. Our finding is different from the findings of a previous study [81] which revealed that negative side effects, such as caused by chronic inflammation, are known to be improved using the routine exercise. Individual studies [59, 80] reported a significant reduction in high sensitivity C-reactive protein, while Roos et al. [63], reported no significant effect on the outcome. Pedro et al. [62], also reported that there was no significant effect on IL-5, 4, 10 that were assessed. Overall, 25% (i.e. two out of eight) of the studies on the effects of exercises on inflammatory biomarkers reported no significant effect.

For the cardiopulmonary outcomes: Ten of the fourteen studies that assessed for VO2 max were included in the meta-analyses [36, 65, 67, 69–74, 76], which revealed that there are a potentially clinically significant improvements in VO_2_ max among the exercisers when compared with the control group. These findings are similar to findings of previous reviews conducted by Jaggers and Hand [81], Nixon et al. [82], O’Brien et al. [83], O’Brien et al. [84], and O’Brien et al. [85]. Individual studies that evaluated other cardiopulmonary outcomes such as the slope/intercept values for heart-Rate workload relationship [68], WRmax [75], and FEV1 [66], reported a significant improvement in the values of the assessed outcomes. Overall, exercise appears to have a beneficial effect on adults living with HIV with cardiopulmonary dysfunction.

This review includes 21 randomized controlled trials and one case-control trial comparing the effect of different exercise interventions to non-exercising control or an alternative intervention. Eight of the included studies used aerobic exercises as intervention while three of the studies employed the use of resistant exercise only. Eleven of the included studies in this review involved concurrent exercise training (combined resistive and aerobic exercise interventions) thus underlining an increasing trend of combined exercise interventions in the literature [36, 59–62, 68–70, 73, 76, 77]. The recommended dosage of aerobic exercise recorded to be effective in this review was based on the exercise Frequency, Intensity, Time and Type (FITT) principle: exercise frequency of 3–5 sessions/week; exercise intensity: 45–80% age-predicted maximal heart rate (APMHR),40–65% of VO2max/peak, 40–75% of heart rate (HR), 50–80% of lactic acidosis threshold (LAT) work rate; exercise time: 15–60 min; and exercise type: prolonged endurance exercise involving large muscle groups, such as those used in walking, running, cycling. The resistant exercises involve exercise frequency of 3–5 sessions/week; exercise intensity: 60–80% of one repetitive maximum (1-RM), 2–5 sets of 8–15 reps. Collectively, the results of this review are complementary to other previous review involving either or both of the interventions highlighting the benefits of exercise for people living with HIV.

### Quality of evidence

Meta-analyses were limited due to incomplete data outcome reporting, low sample size, comparison groups and variation in outcome measured. Studies included in this review demonstrated a high risk of performance bias due to the inability to blind participants and exercise supervisors in the exercise intervention. Studies that blinded the assessors of outcome were limited resulting in a high risk of assessor bias. Furthermore, there was a high risk of attrition bias evident in the incomplete data and a dropout rate of > 15% was viewed in many of the studies together with the failure to conduct an ‘Intention-to-treat’ analysis. These subsequently brought about a moderate quality rate in many of the included studies using the GRADE ratings for quality of evidence.

## Conclusions

### Implications for practice

There is evidence that cardiopulmonary function is significantly improved in patients with HIV infection by engaging in either aerobic exercises (jogging, brisk walking, stretching exercises, cycling, treadmill, and cross trainer - for 30–60 min, 3–5 times/week, at 40–80% _max_HR, or 50–80% VO_2_ or 50% of LAT work rate or heavy aerobic – 50% difference between LAT and VO_2max_ on cycle ergometer) or resistance exercises (1–6 free weight exercises, 3 times/week, at 60–80% of 1 RM, 3–4 sets of 8–12 RM, or 60–80% V_2_ peak and muscular endurance, for 30–60 min) or a combination of both exercises [aerobic exercise - 15 -20mins, at 50–70% of HRrest, plus resistance exercises (free weight and machine), 2–3 sets of 8–12 RM, for 3–5 times/week]. The findings from this study are very important because available evidence [86] suggests that co-morbidities that are associated with degeneration in HIV conditions are worsened by malnutrition, lack of physical exercise and restriction in social participation leading to multi-system (neurological, musculoskeletal, cardiopulmonary and metabolic) impairments. These conditions could minimisze functional mobility with adverse impact on socialiszation resulting in restricted social participation at the community level. Therefore, the evidence from this study recommends physical exercises (specifically either aerobic exercises, resistance exercises or a combination of both) as an effective clinical tool for addressing some of the cardiopulmonary health challenges of PLWHA such that improved management of these disease symptoms using physical exercises might improve physical and social functioning at no extra financial cost, and with little or no side effects to the PLWHA. However, there is no evidence that these interventions have significant effects on inflammatory biomarkers in patients living with HIV. The interpretation of this finding should not be seen as ‘No evidence of effect’ because the individual trial studies did not attain sufficient power to detect treatment effects due to the small sample sizes.

### Implication for research

The research found more published studies that assessed the combination of both aerobic and resistance interventions with the inclusion of flexibility exercises and home-based pedometer walking program [63]. There was also existing co-interventions such as Education [63], diets [74], and counseling [66]. Evidence regarding the effects of exercise on the specified outcomes, especially for the biomarkers, is limited by the high rate of withdrawal from the study and non-adherence on the part of the participants. Moreover, meta-analyses could not be conducted on some of the parameters such as Interleukin-8, 10 hs-CRP and Forced Expiratory Volume in 1 sec as a cardiopulmonary outcome as a result of the limited number of available clinical trials especially RCTs.

The study population included both younger and older adults both male and female without considering the variation in the age of participants which could possibly influence the results. In addition, the inclusion of a pilot study with high risk of bias can possibly influence the evidence of the research. Therefore, further studies should address the limited number of RCTs and the weaknesses of the available studies as already mentioned above, otherwise it will be difficult to reach a scientific conclusion that will guide practice on the effects of exercises on such parameters as Interleukin-8, 10, hs-CRP and Forced Expiratory Volume in 1 sec as cardiopulmonary outcome measures.

### Limitations of study

1. The use of an active control group resulted in a difficulty in determining the specific values of the outcomes versus the usual care or nothing.
2. Inability to conduct meta-analyses for all the outcomes assessed by the included studies due to the limitation of incomplete outcome reporting. This limited the evidence of findings.

## Additional files

Additional file 1: Search strategy in PubMed for inflammatory biomarkers. The *MESH* terms used to search the Pubmed database for evidence of the effects of physical exercises on inflammatory biomarkers in HIV conditions. (DOCX 14 kb)

Additional file 2: Search strategy in PubMed for cardiopulmonary function. The *MESH* terms used to search the Pubmed database for evidence of the effects of physical exercises on cardiopulmonary function in HIV conditions. (DOCX 14 kb)

Additional file 3: Search strategy in Cochrane library for inflammatory biomarkers. The *MESH* terms used to search the Cochrane library database for evidence of the effects of physical exercises on inflammatory biomarkers in HIV conditions. (DOCX 13 kb)

Additional file 4: Search strategy in Cochrane library for cardiopulmonary function. The *MESH* terms used to search the Cochrane library database for evidence of the effects of physical exercises on cardiopulmonary function in HIV conditions. (DOCX 13 kb)

Additional file 5: Quality of evidence and definition. A definition of the different categories of quality of evidence applied in grading the studies included in the research. (DOCX 19 kb)

## Abbreviations

1-RM: One repetitive maximum
AMED: Allied and Complementary Medicine Database
APMHR: Age-predicted maximal heart rate
ART: Highly Active Antiretroviral therapy
BMI: Body mass index
CINAHL: Cumulative Index to Nursing and Allied Health Literature
CON: Control
CRP: C-reactive protein
CVD: Cardiovascular disease
EMBASE: Excerpta Medica database
EX: Exercise
FEV_1_: Forced expiratory volume at the first second
FITT: Frequency, Intensity, Time and Type
GRADE: Grading of Recommendations Assessment Development and Evaluation
HIV: Human immune deficiency virus
HR: Heart rate
HR_5min_: Submaximal analysis of HR
Hs-CRP: High sensitivity C-reactive protein
IFN-ϒ: Interferon gamma
IL-10: Interleukin-10
IL-18: Interleukin 18
IL-2: Interleukin-2
IL-4: Interleukin-4
IL-5: Interleukin-5
IL-6: Interleukin-6
LAT: Lactic acidosis threshold
MeSH: Medical subject heading
MOS: Medical outcomes study
NHREC: Nigeria Health Research Ethics Committee
O_2_ pulse: Oxygen pulse
OIs: Opportunistic illnesses
PEDro: Physiotherapy Evidence Database
PLWH: People living with HIV/AIDS
PRG: Progressive resistance exercise group
PRISMA: Preferred Reporting Items for Systematic Reviews and Meta-analyses
PROSPERO: International Prospective Register of Systematic Reviews
QoL: Quality of life
RCTS: Randomized control trials
RevMan: Review Manager
ROB: Risk of bias
ROS: Reactive oxygen species
SCD14: Soluble CD14
TNF- α: Tumour necrosis factor alpha
TV: Tidal volume
VE: Ventilation
VO_2_ max: Maximum Oxygen uptake/Consumption
VO_2_ peak: Peak Oxygen uptake
VO2 sub: Submaximal oxygen uptake
Vpeak: Peak running speed
WHS: Women’s Health Study
WRmax: Maximal work rate
