# Supplementary material for: Effects of physical exercises on inflammatory biomarkers and cardiopulmonary function in patients living with HIV: a systematic review with meta-analysis
Source: BMC Infect Dis. 2019 Apr 29;19:359. doi: 10.1186/s12879-019-3960-0 (PMC6489236; doi:10.1186/s12879-019-3960-0)
Supplement: Supplementary file 1 — Search strategy in PubMed for inflammatory biomarkers. The MESH terms used to search the Pubmed database for evidence of the effects of physical exercises on inflammatory biomarkers in HIV conditions. (DOCX 14 kb) [file 12879_2019_3960_MOESM1_ESM.docx]

Additional file 1

Search Strategy in PubMed for inflammatory biomarkers

| CONCEPT | SEARCH TERMS |
| --- | --- |

Population 1. HIV

2. HIV-1

3. HIV-2

4. Human Immunodeficiency Virus

5. AIDS

6. Acquired Immunodeficiency Syndrome

7. Retroviridae

8. Retrovirus

9. Seropositive

10. 1 OR 2 OR 3 OR 4 OR 5 OR 6 OR 7 OR 8 OR 9

Intervention 11. Physical Exercise

12. Exercise training

13. Exercise therapy

14. Aerobic exercise

15. Resistance exercise

16. Physical activity

17. Strength training

18. Endurance training

19. Isometric exercise

20. 11 OR 12 OR 13 OR 14 OR 15 OR 16 OR 17 OR 18 OR 19

Design 21. Randomised Contorlled Trials

22. Clinical Trials

23. Random Allocation

24. Control groups

25. 21 OR 22 OR 23 OR 24

Outcome 26. Inflammation

27. Inflammatory biomarkers

28. C-reactive protein

29. Interleukin-6

30. Interleukin-8

31. Interleukin-10

32. Tumor Necrosis Factor-alpha

33. Pro-inflammatory cytokines

34. Anti-inflammatory cytokines

35. 26 OR 27 OR 28 OR 29 OR 30 OR 31 OR 32 OR 33 OR 34

36. 10 AND 20 AND 25 AND 35

|  |
| --- |
