# Supplementary material for: Effects of physical exercises on inflammatory biomarkers and cardiopulmonary function in patients living with HIV: a systematic review with meta-analysis
Source: BMC Infect Dis. 2019 Apr 29;19:359. doi: 10.1186/s12879-019-3960-0 (PMC6489236; doi:10.1186/s12879-019-3960-0)
Supplement: Supplementary file 2 — Search strategy in PubMed for cardiopulmonary function. The MESH terms used to search the Pubmed database for evidence of the effects of physical exercises on cardiopulmonary function in HIV conditions. (DOCX 14 kb) [file 12879_2019_3960_MOESM2_ESM.docx]

Additional file 2

Search Strategy in PubMed for Cardiopulmonary function

| CONCEPT | SEARCH TERMS |
| --- | --- |

Population 1. HIV

2. HIV-1

3. HIV-2

4. Human Immunodeficiency Virus

5. AIDS

6. Acquired Immunodeficiency Syndrome

7. Retroviridae

8. Retrovirus

9. Seropositive

10. 1 OR 2 OR 3 OR 4 OR 5 OR 6 OR 7 OR 8 OR 9

Intervention 11. Physical Exercise

12. Exercise training

13. Exercise therapy

14. Aerobic exercise

15. Resistance exercise

16. Physical activity

17. Strength training

18. Endurance training

19. Isometric exercise

20. 11 OR 12 OR 13 OR 14 OR 15 OR 16 OR 17 OR 18 OR 19

Design 21. Randomised Contorlled Trials

22. Clinical Trials

23. Random Allocation

24. Control groups

25. 21 OR 22 OR 23 OR 24

Outcome 26. Cardiopulmonary function

27. Cardiopulmonary fitness

28. Cardiorespiratory function

29. Cardiorespiratory fitness

30. VO2 maximum

31. Aeerobic fitness

32. Oxygen consumption

33. Peak Expiratory Rate

34. Forced Expiratory Volume

35. 26 OR 27 OR 28 OR 29 OR 30 OR 31 OR 32 OR 33 OR 34

36. 10 AND 20 AND 25 AND 35

|  |
| --- |
