# Supplementary material for: Effects of physical exercises on inflammatory biomarkers and cardiopulmonary function in patients living with HIV: a systematic review with meta-analysis
Source: BMC Infect Dis. 2019 Apr 29;19:359. doi: 10.1186/s12879-019-3960-0 (PMC6489236; doi:10.1186/s12879-019-3960-0)
Supplement: Supplementary file 3 — Search strategy in Cochrane library for inflammatory biomarkers. The MESH terms used to search the Cochrane library database for evidence of the effects of physical exercises on inflammatory biomarkers in HIV conditions. (DOCX 13 kb) [file 12879_2019_3960_MOESM3_ESM.docx]

Additional file 3

Search Strategy in Cochrane library for inflammatory biomarkers

| CONCEPT | SEARCH TERMS |
| --- | --- |

Population 1. HIV

2. AIDS

3. HIV- Seropositivity

4. 1 OR 2 OR 3

Intervention 11. Physical Exercise

12. Exercise training

13. Exercise

14. Aerobic exercise

15. Resistance exercise

16. Physical activity

17. Isometric exercise

18. 11 OR 12 OR 13 OR 14 OR 15 OR 16 OR 17

Design 18. Randomised Contorlled Trials

19. Clinical Trials

20. Random Allocation

21. Control groups

22. 21 OR 22 OR 23 OR 24

Outcome 23. inflammation

24. inflammatory biomarkers

25. Tumoer Necrosis factor-alpha

26. interleukin-6

37. interleukin-8

31. inteleukin-10

32. 26 OR 27 OR 28 OR 29 OR 30 OR 31

33. 4 OR 18OR 22 OR 32

|  |
| --- |
