# Supplementary material for: Effects of physical exercises on inflammatory biomarkers and cardiopulmonary function in patients living with HIV: a systematic review with meta-analysis
Source: BMC Infect Dis. 2019 Apr 29;19:359. doi: 10.1186/s12879-019-3960-0 (PMC6489236; doi:10.1186/s12879-019-3960-0)
Supplement: Supplementary file 4 — Search strategy in Cochrane library for cardiopulmonary function. The MESH terms used to search the Cochrane library database for evidence of the effects of physical exercises on cardiopulmonary function in HIV conditions. (DOCX 13 kb) [file 12879_2019_3960_MOESM4_ESM.docx]

Additional file 4

Search Strategy in Cochrane library for Cardiopulmonary function

| CONCEPT | SEARCH TERMS |
| --- | --- |

Population 1. HIV

2. AIDS

3. HIV- Seropositivity

4. 1 OR 2 OR 3

Intervention 11. Physical Exercise

12. Exercise training

13. Exercise

14. Aerobic exercise

15. Resistance exercise

16. Physical activity

17. Isometric exercise

18. 11 OR 12 OR 13 OR 14 OR 15 OR 16 OR 17

Design 18. Randomised Contorlled Trials

19. Clinical Trials

20. Random Allocation

21. Control groups

22. 21 OR 22 OR 23 OR 24

Outcome 23. Cardiopulmonary function

24. Cardiopulmonary fitness

25. Cardiorespiratory function

26. Cardiorespiratory fitness

37. VO2 maximum

31. Aerobic fitness

32. 26 OR 27 OR 28 OR 29 OR 30 OR 31

33. 4 OR 18OR 22 OR 32

|  |
| --- |
|  |
