# Supplementary material for: Effects of physical exercises on inflammatory biomarkers and cardiopulmonary function in patients living with HIV: a systematic review with meta-analysis
Source: BMC Infect Dis. 2019 Apr 29;19:359. doi: 10.1186/s12879-019-3960-0 (PMC6489236; doi:10.1186/s12879-019-3960-0)
Supplement: Supplementary file 5 — Quality of evidence and definition. A definition of the different categories of quality of evidence applied in grading the studies included in the research. (DOCX 19 kb) [file 12879_2019_3960_MOESM5_ESM.docx]

**Additional file 5**

**Quality of evidence and definition (*adapted from Guyatt et al. 2008*)**

Grading

High quality Evidence derived from many studies of high quality such that further research is very unlikely to change our confidence in the estimate of effect.

Moderate Evidence derived from mostly studies of moderate quality and/or few high quality

Quality studies such that further research is likely to hhhave an important impact on our confidence in the estimate of effect and may change the estimate.

Low quality Evidence derived from mostly low quality studies and/or few studies of moderate quality such that further research is very likely to have an important impact on our confidence in the estimate of effect and is likely to change the estimate

Very Low All evidence derived from the studies of low quality studies such that any

Quality estimate of effect is very uncertain.
